# Supplementary material for: Enhancing Progestin Therapy with a Glucagon-Like Peptide 1 Agonist for the Conservative Management of Endometrial Cancer
Source: Cancers (Basel). 2025 Feb 10;17(4):598. doi: 10.3390/cancers17040598 (PMC11853405; doi:10.3390/cancers17040598)
Supplement: Supplementary file 1 [file cancers-17-00598-s001.zip › cancers-3363677-supplementary.pdf]

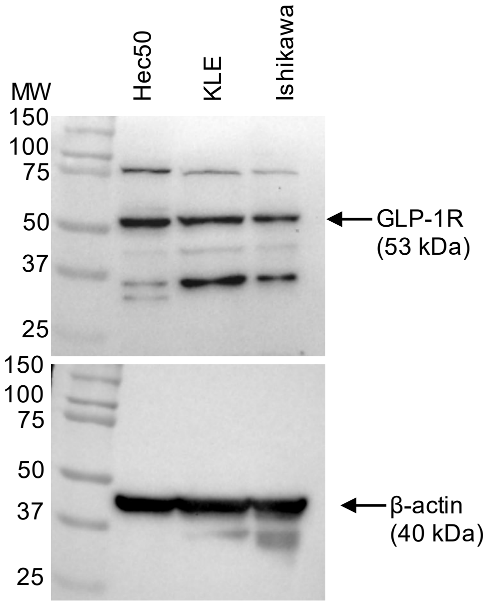


**SDS-PAGE & transfer conditions:**
Load 20 µg lysate per cell line in 10% gel
SDS-PAGE: 2 hrs, 150 V @ room temperature
Transfer overnight (wet) at 4°C, nitrocellulose membrane

**GLP-1R blot**
Block in 5% Milk/TBST for 1 hr
Primary GLP-1R (#bs-1559R, Bioss Antibodies): 1:1000 dilution in 5% BSA, o/n @ 4°C
Wash in TBST x 3 (20 min each)
Secondary (anti-rabbit-HRP): 1:10,000 dilution in 5% BSA 1 hr @ RT
Wash in TBST x 3, 20 min each wash
Develop: SuperSignal West Pico Plus (Thermo), 152 second exposure

**β-actin blot** (using same membrane used for GLP-1R blot)
Block in 5% Milk/TBST for 15 min
Primary β-actin: 1:5000 dilution in 5% Milk/TBST 1 h RT
Wash in TBST x 3 10 min each
Secondary (anti-mouse HRP): 1:10,000 dilution in 5% BSA 1 hr @ RT
Wash in TBST x 3, 10 min each wash
Develop: SuperSignal West Pico Plus (Thermo), 3 sec exposure
